# Correlation between the *In Vitro* Functionality of Stored Platelets and the Cytosolic Esterase-Induced Fluorescence Intensity with CMFDA

**DOI:** 10.1371/journal.pone.0138509

**Published:** 2015-09-21

**Authors:** Jiexi Wang, Xiaoyang Yi, Minxia Liu, Qian Zhou, Suping Ren, Yan Wang, Chao Yang, Jianwei Zhou, Ying Han

**Affiliations:** 1 Beijing Institute of Blood Transfusion, Academy of Military Medical Sciences, Beijing, China; 2 Beijing Red Cross Blood Center, Beijing, China; 3 The Second Artillery General Hospital PLA, Beijing, China; University Hospital Medical Centre, GERMANY

## Abstract

It has been hypothesized that the cytosolic esterase-induced fluorescence intensity (CEIFI) from carboxy dimethyl fluorescein diacetate (CMFDA) in platelets may related to platelet functions. In the present study, we measured the change of CEIFI in platelets during storage, and examined the correlations of CEIFI with the in vitro functionality of stored platelets, including the ADP-induced aggregation activity, hypotonic shock response, expression of CD62P as well as platelet apoptosis. The CEIFI of fresh platelets, when tested at 10 μM CMFDA, the mean fluorescence intensity index (MFI) was 305.9 ± 49.9 (N = 80). After 1-day storage, it was 203.8 ± 34.4, the CEIFI of the stored platelets started to decline significantly, and reduced to 112.7 ±27.7 after 7-day storage. The change in CEIFI is highly correlated to all four functional parameters measured, with the correlation coefficients being 0.9813, 0.9848, -0.9945 and -0.9847 for the ADP-induced aggregation activity, hypotonic shock response (HSR), expression of CD62P and platelet apoptosis respectively. The above results show that the CEIFI measurement of platelets represents well the viability and functional state of in vitro stored platelets. This may be used as a convenient new method for quality evaluation for stored platelets if this result can be further validated by the following clinical trials.

## Introduction

Platelet transfusion is the most effective treatment for hemorrhage patients with severely reduced platelet counts and with damaged or impaired platelet functions [1]. The quality of platelets directly affects the therapeutic effects of clinical transfusion. The Food and Drug Administration of the United States stipulates that platelets can be stored for five days at 22°C under horizontal vibration, but the method and threshold of functional evaluation of platelets acquired singly during storage periods have not been specified. There are several assays for measuring certain aspects of platelet function, including the assessment of platelet adhesion, activation and aggregation, however, all of them have measuring certain aspects. Presently the functional evaluation of platelet quality could not be made during storage because in vitro indicators that can accurately reflect the functional states and therapeutic efficacy of platelets have yet to be established and adopted. Therefore, any new assay that can better reflect the functional state and/or transfusion efficacy would be of great value.

CMFDA pass freely through cell membranes. CMFDA is colorless and non-fluorescent. But once inside the cell, these molecules may become membrane impermeable. CMFDA molecules contain a chloromethyl group that can react with thiols, probably via a glutathione S-transferase–mediated reaction [2–4]. The impermeable products of the chloromethyl coumarins have excellent retention, strong fluorescence and relatively uniform cytoplasmic staining, making these derivatives potentially useful for correcting motion artifacts in imaging. Cytosolic esterases can also cleave off the acetate groups in the CMFDA molecules, releasing a brightly fluorescent product [5]. The CEIFI of cells stained by CMFDA correlates well with cell viability and function [6]. Nowadays CEIFI of cells stained by CMFDA is often used for evaluating cell viability due to the convenience, accuracy and sensitivity. For example, it is used to assess the quality of collected semen in mammals and to measure sperm motility in vitro after storage [7–8]. In the present study, we have investigated the utility of CEIFI of platelets stained by CMFDA as a physiological indicator for the functional state of stored platelets. The objective of the present study is to examine how well CEIFI of platelets is correlated to the functional aspects of stored platelets in vitro such as aggregation, activation, apoptosis and anti-hypotonic shock response.

## Materials and Methods

### Reagents and Antibodies

CellTracer^TM^ Green CMFDA(5-chlorome-thylfluorescein) was obtained from life technologies^TM^ (Molecular Probes, Eugeon, Oregon, USA). Adenosine diphosphate (ADP) was obtained from Sigma (St. Louis, Missouri, USA). Mouse IgG1 κ Iso control FITC and Anti-human CD62P-FITC antibody was obtained from eBiosciences Inc. (San Diego, CA). Annexin V-FITC Kit was obtained from KeyGEN BioTECH (Nanjing, China).

### Collection and Treatment of Platelet Samples

The study was approved by the Ethics Committee of the Academy of Military Medical Sciences and all aspects of the study comply with the Declaration of Helsinki. Ethics Committee of the Academy of Military Medical Sciences specifically approved that not informed consent was required because data were going to be analysed anonymously. All platelet rich plasma (PRP, apheresis platelets) samples were collected by the Beijing Red Cross Blood Center from healthy adults with ACD as the anti-coagulant (Baxter Single Needle Platelet Collection, A2-0766-001 Rev, Baxter® Amicus TM Separator). The age of eighty PRP donors was between 23 and 55, with a mean of 37 and a male/female ratio of 7/3. The pH value was 7.26±0.08, platelet counts value was (1018.4±44.9)×10^9^ platelets/L, and platelet volumes (MPV) was 7.08±0.46(fl). The aliquots of 8ml PRP sample were placed into a plastic centrifuge tube stored in a platelet thermostatic storage box (XHZ-IIIA, Suzhou Medical Instrument Factory, Suzhou, China) for 7 days. Storage temperature was maintained at 22 ± 2°C. Platelet samples were taken daily for analysis.

### Measurement of Cytosolic Esterase Induced Fluorescence in Platelets

Samples of resting platelets and platelets activated by 100μM ADP were adjusted to the platelet concentration of 3.5~3.8 × 10^8^/L with autologous plasma, and incubated for 30 minutes at 37°C in 10 μM CMFDA. After incubation, the cytosolic esterase-induced fluorescence of platelets was measured by laser confocal microscopy (LSM 510, Zeiss, German) and also photograph was taken.

Aliquots of 80 μl PRP sample with concentration of 3.5~3.8 × 10^8^/L were incubated for 30 minutes with 20 μl CMFDA with the final concentration of 10μM. After incubation, the samples were diluted with 500 μl PBS (pH 7.4) for the CEIFI analysis with flow cytometry (Cytomics FC 500 Beckman Coulter, USA), and the MFI was used to represent platelet CEIFI.

### Measurement of Platelet Aggregation Activity

Platelet aggregation was measured by light transmission aggregometry (LTA, Chrono-log Model 700, USA) in response to 5 μM ADP. The cell concentration was 200–300 K/μl, and the corresponding platelet poor plasma (PPP) was used as the control. An aliquot of 500-μl PRP was added into a reaction cup with continuous stirring at 37°C, and then aggregation agonists ADP was added to the PRP sample. After incubation for 6 to 9 min, platelet aggregation activity was measured, and the maximum aggregation rate was used to represent platelet aggregation activation.

### Measurement of Platelet Resistance to HSR

The PRP was diluted with phosphate buffered saline (PBS) and sterile deionized water at a dilution ratio of 1:1, and was then placed in an ultraviolet spectrophotometer (UV-2550, SHIMADZU, Japan) at 37°C. HSR was measured according to dynamic light transmittance, and was calculated with the equation HSR = [(B-C) / (B-A)] × 100, where A is the transmittance of the PBS-diluted platelet samples, B is the maximum transmittance of deionized water diluted platelet samples, and C is the minimum transmittance of deionized water diluted platelet samples.

### CD62P Expression on Platelet Membranes

The CD62P (P-selectin) expression level of platelets during storage were measured by flow cytometry (Cytomics FC500, BECKMAN, New York, USA). Platelets were centrifuged at 1200g for 10 min. Precipitated platelets were re-suspended and diluted in PBS. Aliquots of 80 μl platelet sample (1 million cells) were incubated for 30 min with 20 μl (0.5 μg) anti-human CD62P-FITC antibody. After incubation at room temperature in dark, samples were diluted with 500 μl PBS (pH 7.4) for analysis with flow cytometry.

### Phosphatidylserine (PS) Externalization of Platelets

PS externalization of platelets was detected by using an Annexin V-FITC Kit. PRP was centrifuged at 1200 × g for 10 min at RT, and the pellet was washed twice with phosphate buffer. Washed platelets were re-suspended in binding buffer to a final cell density of 3 × 10^8^/ml. Then 5 μl of Annexin V-FITC was added to a platelet sample of 5 μl, mixed evenly, and incubated at room temperature in the dark for 15 min. PS externalization of platelets was detected by flow cytometry in 1 hour. The extent of platelet apoptosis was represented by the expression of PS.

### Statistical Analysis

The experimental data were expressed as mean ± S.D. Each experiment was carried out at least three times. Statistical comparison among the data (means) were performed by one-way analysis of variance (ANOVA), followed by post-hoc Dunnett’s test. A *P*-value of less than 0.05 was considered statistically significant. The correlation coefficients between the CEIFI and in vitro functional parameters of platelets were calculated by using the Pearson product-moment correlation coefficient analysis(r or Pearson’s r).

## Results

### The CEIFI of Platelets Decreases Significantly During 7 Days Storage

**Fig 1:**
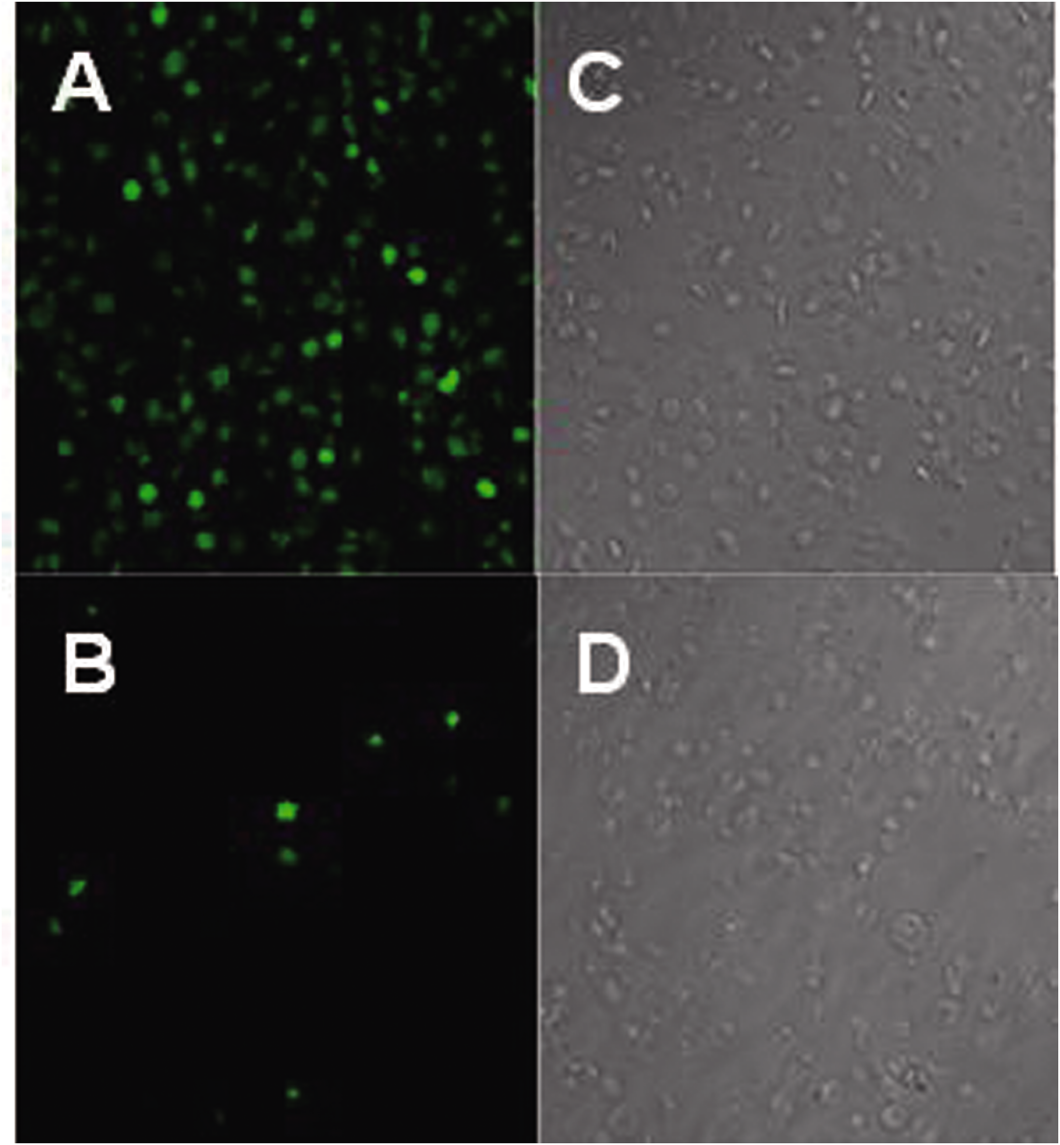
The difference in cytosolic esterase-induced fluorescence with CMFDA between resting and activated human platelets. The activity of cytosolic esterase-induced fluorescence reaction is visualized with CMFDA. The fluorescence of resting platelets was clearly visible in laser confocal microscopy, whereas activated platelets release no or little fluorescence. A, C: Resting platelets; B, D: Activated platelets (activated by 100μM ADP); A, B: green fluorescence; C, D: bright field.

**Fig 2:**
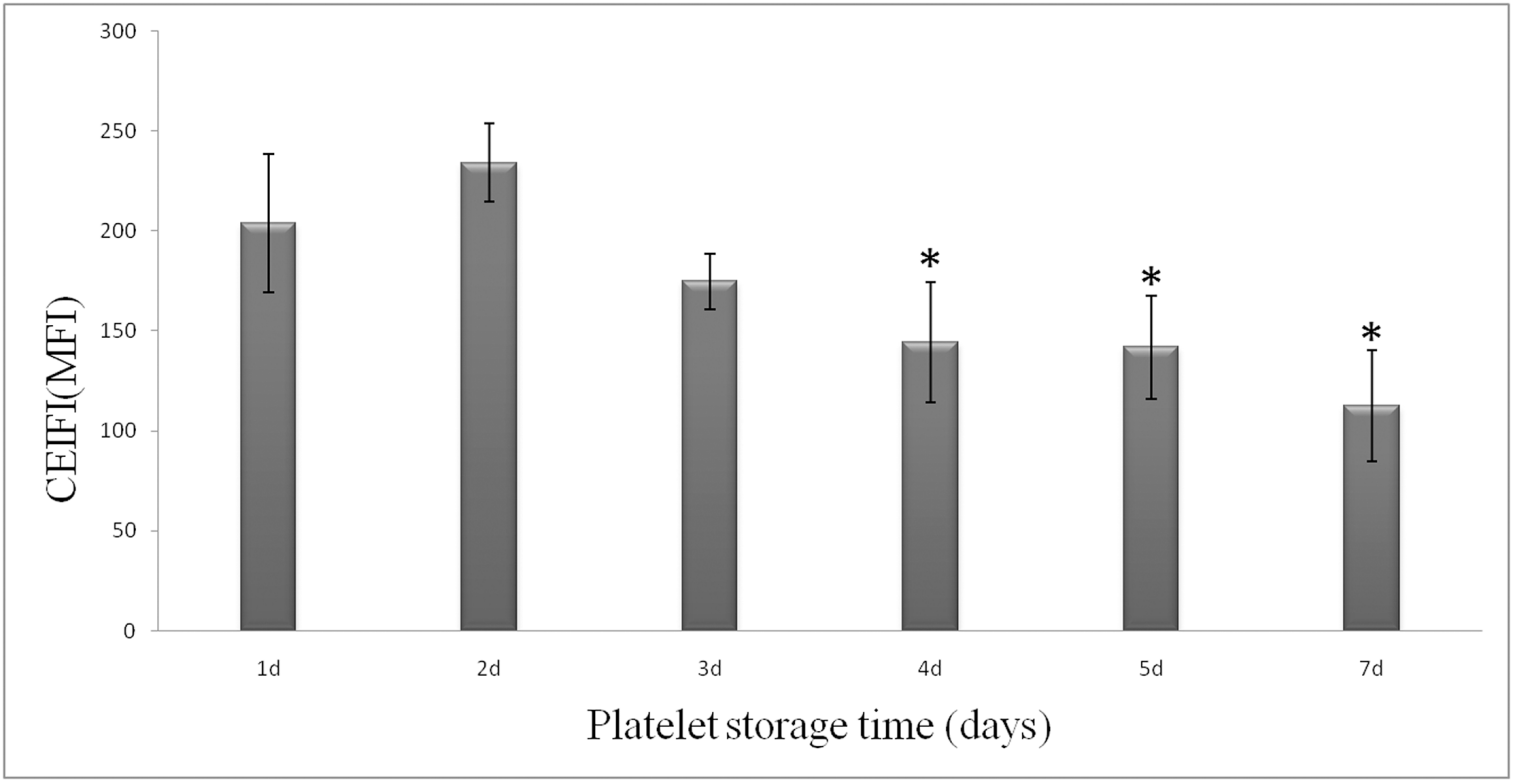
The change of cytosolic esterase-induced fluorescence activity in platelets during 7-day storage at 22 ± 2°C. The activity of cytosolic esterase-induced fluorescence in platelets is measured with CMFDA. *P <0.05 as compared with platelets stored for 1 day.

The observation with laser confocal microscopy on CMFDA-stained platelets shows that nearly all the resting platelets release bright green fluorescence, whereas the ADP activated platelets release no or little fluorescence (Fig 1). The cytosolic esterase induced fluorescence release reaction of platelets stained by CMFDA is a complex chemical reaction occurring within the cell, which requires a complete cellular structure and function as a basis for the reaction occurrence. Once platelet activated by ADP, the platelets loss vitality and function, therefore, the ADP activated platelets release no or little fluorescence. This result demonstrated that visualized fluorescence change within laser confocal microscopy and the platelet CEIFI correlates well with cell viability and function. The MFI increases from 62.5 ± 9.1 to 209.9 ± 65.3 as the concentration of CMFDA increases from 1 μM to 10 μM (data not shown). For the platelet storage study, the concentration of CMFDA was at 10 μM, the MFI for 1-day storage platelet units was determined to be 203.8 ± 34.4. Fig 2 shows that the CEIFI of platelets decreases significantly after storage. After 7-day storage, the MFI of platelets decreases by ~60% down to 112.7±27.7. There were remarkable differences in platelet CEIFI activity on the first and seventh days (*P* <0.05). Thus the CEIFI change of platelets stained with CMFDA correlate with the metabolic state and viability of platelets during storage.

### The Change of Platelet CEIFI and these Four Functional Parameters are Highly Correlated During 7 Days Storage

**Fig 3:**
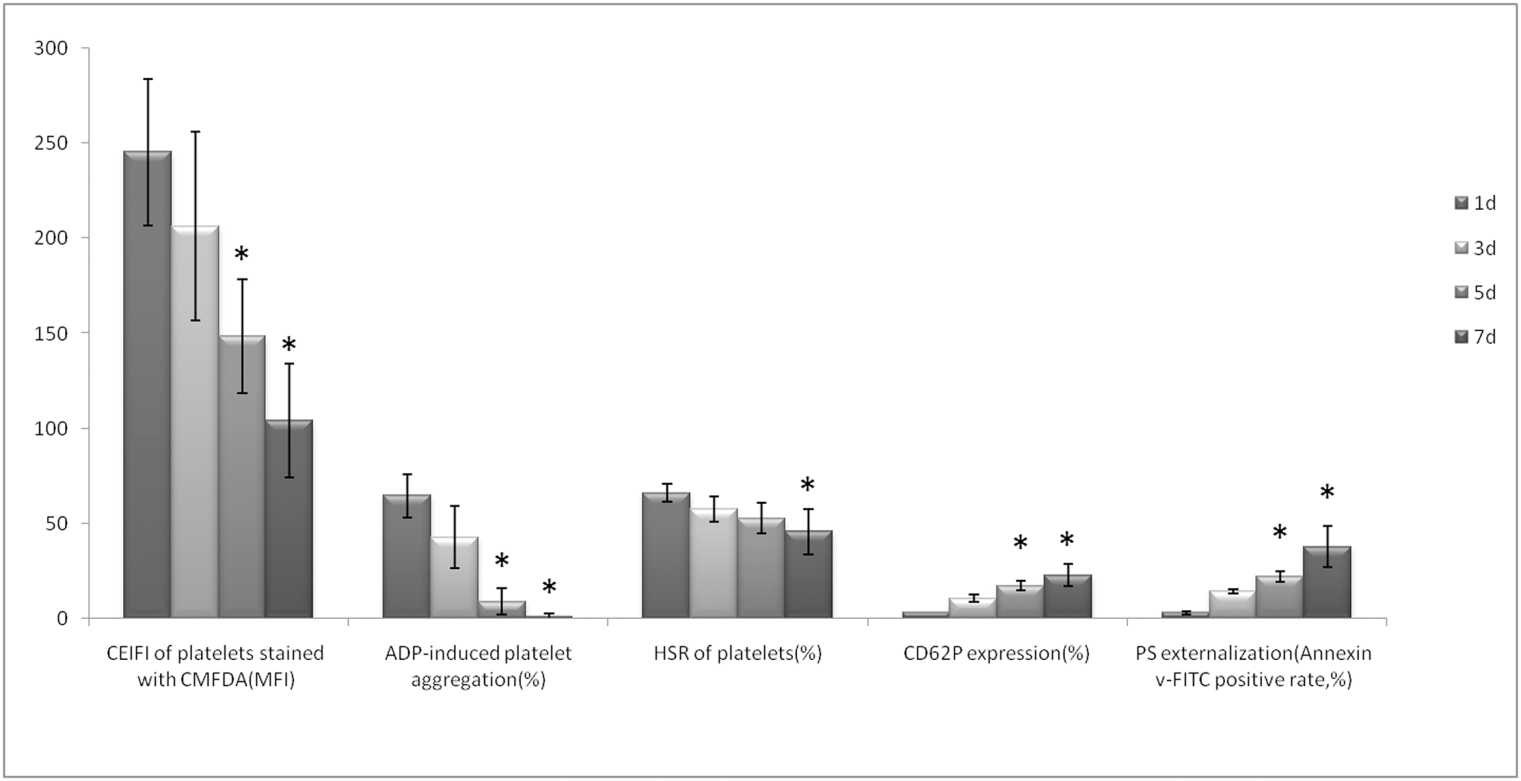
- Changes of CEIFI, aggregation activity, HSR, CD62P expression and PS externalization in platelets during 7-day storage at 22 ± 2°C. Data are the mean ± S.D. *P <0.05 as compared with platelets stored for 1 day.

**Fig 4:**
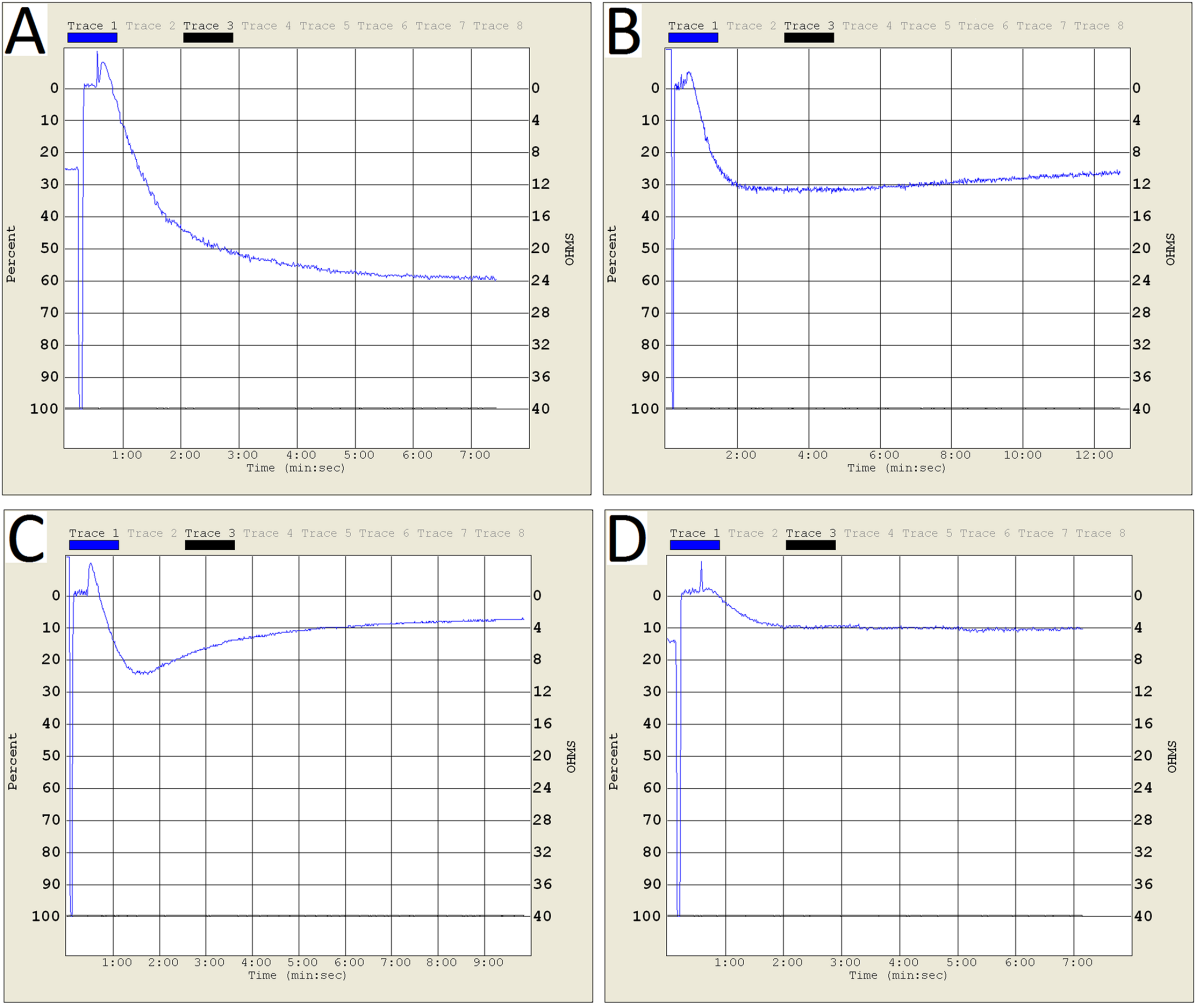
Aggregation curves of platelets during 7-day storage at 22 ± 2°C. A: platelets stored for 1 day. B: platelets stored for 3 days. C: platelets stored for 5 days. D: platelets stored for 7 days.

**Fig 5:**
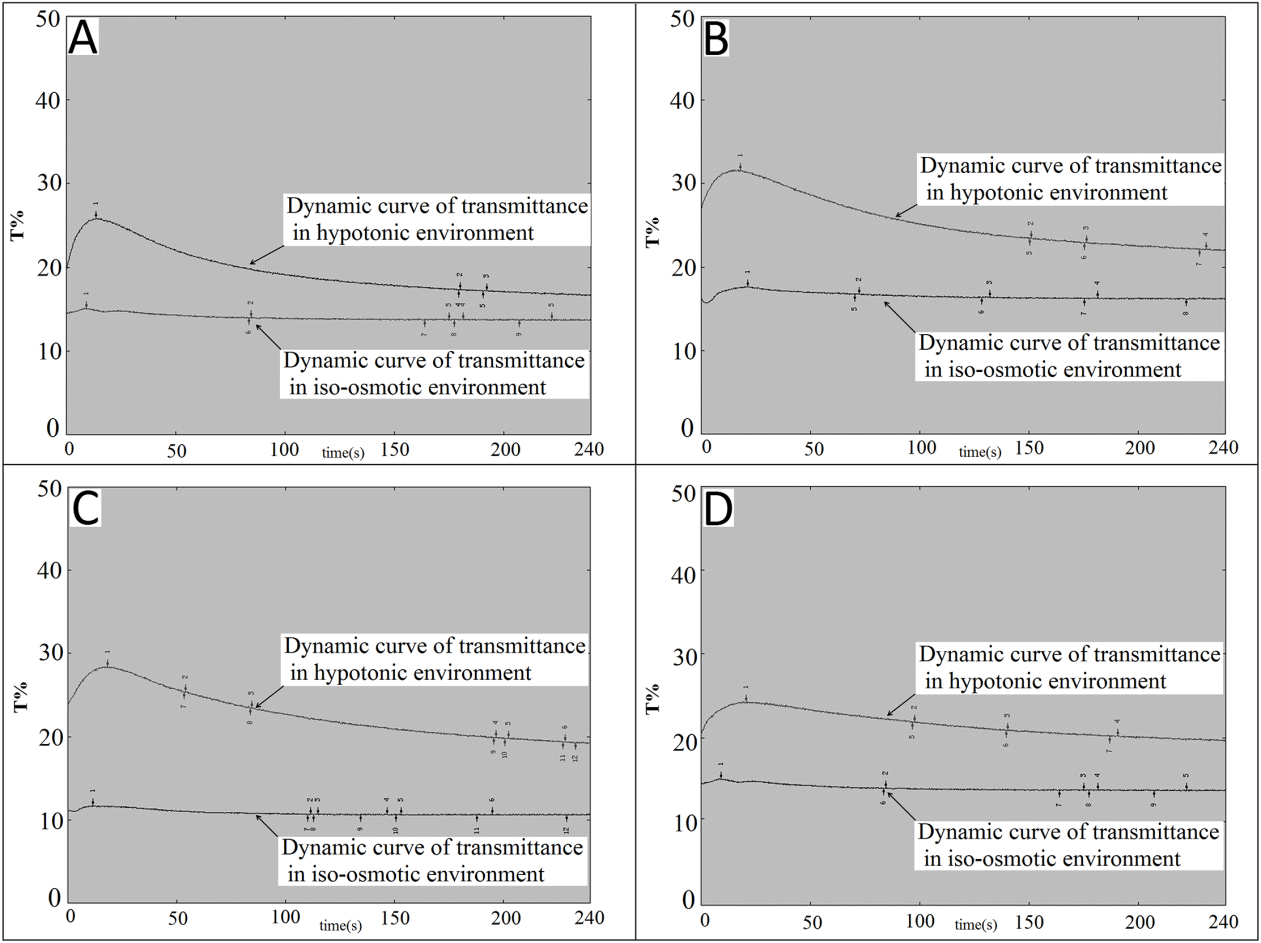
HSR curves of platelets during 7-day storage at 22± 2°C. A: platelets stored for 1 day. B: platelets stored for 3 days. C: platelets stored for 5 days. D: platelets stored for 7 days.

**Fig 6:**
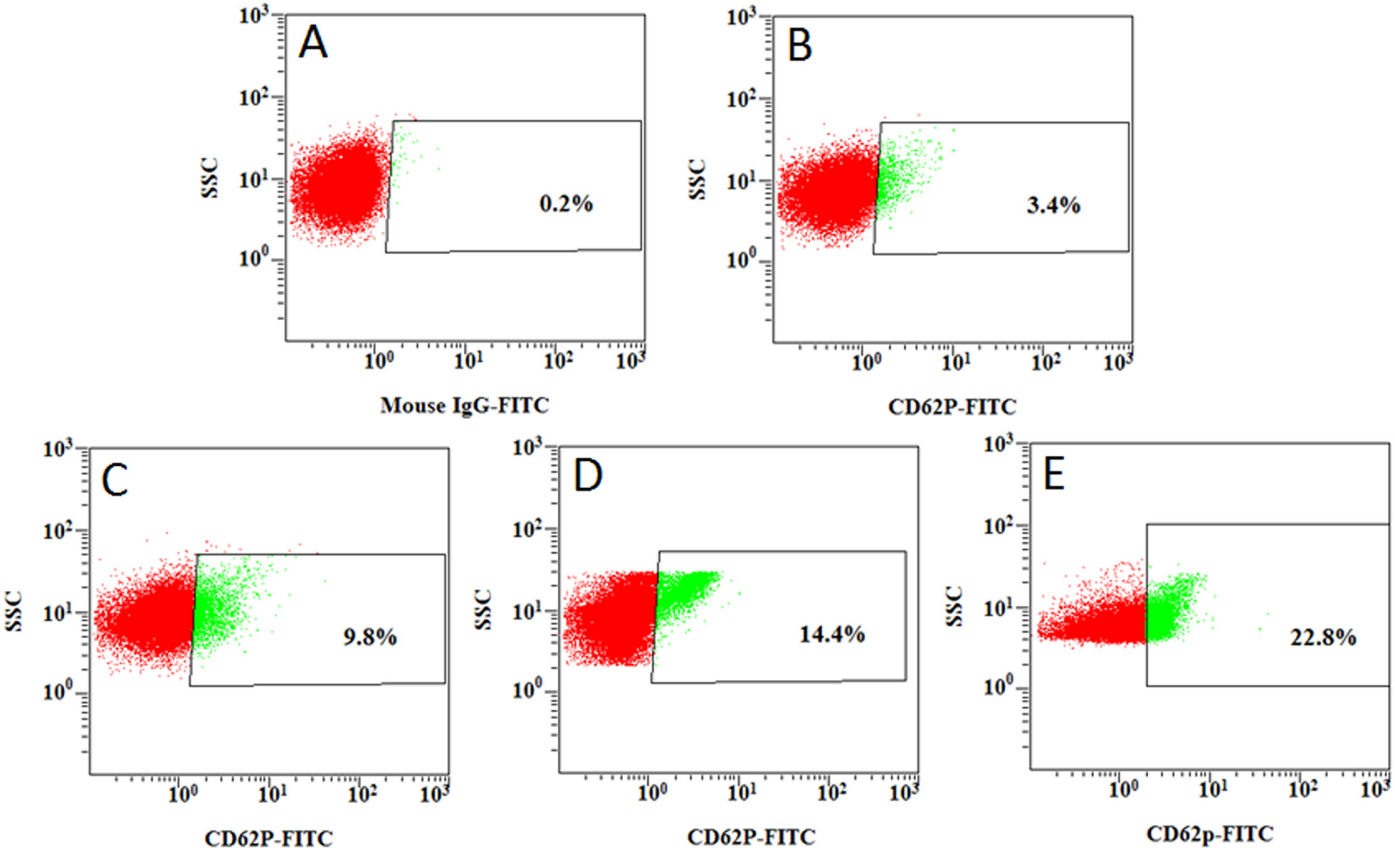
CD62P expression percentage on membrane of platelets during 7-day storage at 22± 2°C. A: Mouse IgG1 κ Iso control. B: platelets stored for 1 day. C: platelets stored for 3 days. D: platelets stored for 5 days. E: platelets stored for 7 days.

**Fig 7:**
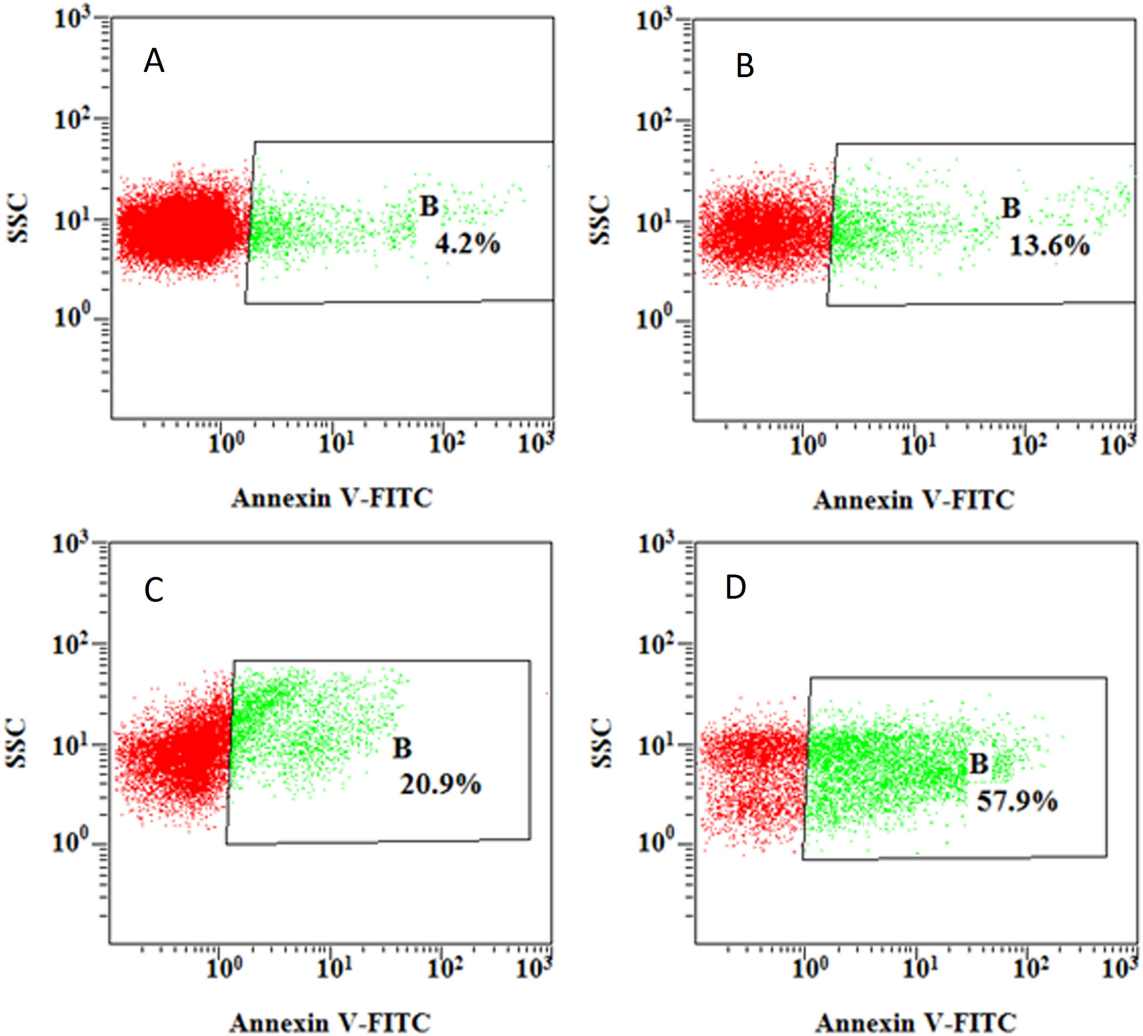
PS externalization of platelets during 7-day storage at 22 ± 2°C. A: platelets stored for 1 day. B: platelets stored for 3 days. C: platelets stored for 5 days. D: platelets stored for 7 days.

**Fig 8:**
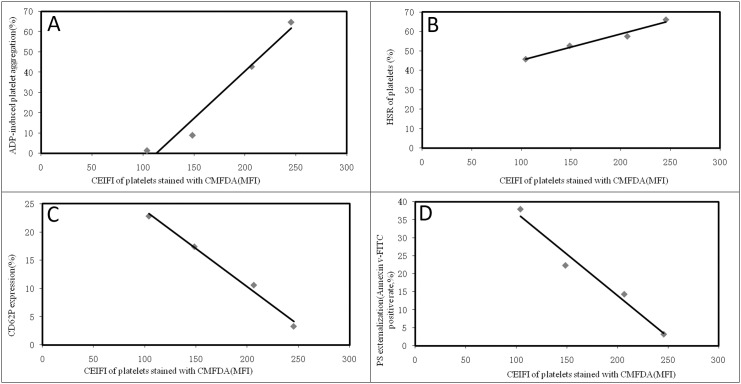
Correlation of platelet CEIFI with functional parameters of platelets during 7-day storage. A: Correlation to the ADP-induced aggregation activity, r = 0.9813. B: Correlation to the HSR activity, r = 0.9848. C: Correlation to the expression percentage of CD62P on platelet membrane, r = -0.9945. D: Correlation to the PS externalization percentage, r = -0.9847.

Changes of platelet function during storage were monitored in vitro via the measurement of four platelet physiological and biochemical parameters, namely aggregation activity, HSR, CD62P expression and PS externalization (an apoptosis marker) (Fig 3). As expected, the ADP-induced aggregation activity and the HSR activity reduced steadily during the course of 7-day storage at 22±2°C (Figs 4 and 5). There were significant differences in platelet aggregation activity and the HSR activity on the 1 day and the 7 days storage (*P* <0.05). On the other hand, the expression of CD62P on platelet membrane and PS externalization increased significantly (Figs 6 and 7). There were remarkable differences in platelet expression of CD62P and the PS externalization on the 1 day and 7 days storage (*P* <0.05). The change of platelet CEIFI and these four functional parameters are highly correlated. The correlation coefficient (r value) was observed to be 0.9813, 0.9848, -0.9945 and -0.9847 respectively, for the correlation of CEIFI to aggregation activity, HSR activity, CD62P expression and PS externalization (Fig 8).

## Discussion

Platelets have a limited life span of 10–12 days only. The quality of a platelet product is routinely evaluated by in vitro assays which are assumed to be predictive of the viability and function of the platelets after therapeutic transfusion. There are several assays for examining the platelet’s functional state, including the assessment of platelet adhesion, activation and aggregation, etc. However, these in vitro assays have various limitations, including the high cost and great complexity of performing the tests, raising the safety and regulatory concerns with research on humans. There is a need to develop a convenient and more sensitive assay for the quality evaluation of stored platelets. The present work investigated the utility of measuring CEIFI in platelets during storage at 22 ± 2°C. The data confirmed that CEIFI is correlated highly with the in vitro functional integrity of stored platelets, including the ADP-induced aggregation activity, hypotonic shock response, expression of CD62P as well as platelet apoptosis. The CEIFI of platelets appears to be a good indicator of the viability and functional state of stored platelets, and the measurement of CEIFI could be a convenient and sensitive new method for quality evaluation of stored platelets.

Platelet aggregation activity has long been the golden standard for platelet function evaluation, the aggregation of PRP as first described by Born [9]. This method measures platelet aggregation as the increase in light transmittance in a PRP sample following the addition of platelet agonists [10]. The ADP induced platelet aggregation activity reduced rapidly during storage at 22±2°C, and the CEIFI of stored platelets followed a similar trend, with a correlation coefficient of 0.9813 between these two parameters.

The response of platelets to osmotic shock and its relationship to platelet viability were studied by Kim BK et al [11]. The hypotonic shock response, a measurement of light absorbency fluctuation (i.e., the reversal reaction) of human platelet concentrates upon the exposure to hypotonic shock is a complex phenomenon caused by the cell volume contraction which depends on a series of biochemical and enzymatic functions of the platelets (e.g., contractile protein and energy availability). A progressive depression of the reversal reaction after storage at 4°C or 22°C is paralleled to the lowered survivability of the platelets in vivo [12], although there are conflicting results on the correlation with platelet viability [13]. We observed that the platelet resistance to HSR activity showed a slow decreasing trend during consecutively vibration storage at 22 ± 2°C for 7 days (Fig 3). The changes in platelets resistance to HSR activity were also positively correlated with changes in CEIFI of platelets (r = 0.9848).

CD62P is present in platelets, and it is rarely expressed in the resting platelets, but could be quickly released upon platelet activation [14]. The presence of surface CD62P is the ‘gold standard’ marker of terminal platelet activation, and indicates membrane platelet membrane disruption. The expression of CD62P in the platelets membrane increases during 7-day storage at 22 ± 2°C (Fig 3), and negatively correlated with the CEIFI of stored platelets (r = -0.9945). PS externalization is an apoptotic marker [15], which can be detected during platelet storage. The expression rate of PS was negatively correlated to the CEIFI of stored platelets (r = -0.9847).

In conclusion, our study has demonstrated that the platelet's CEIFI measurement is able to well represent the changes in the in vitro functional parameters of stored platelets. CEIFI is positively correlated with the aggregation activity and HSR of stored platelets, and negatively correlated with the expression of P-selectin and PS externalization. These results confirmed the relationship between platelet's CEIFI and platelet function in vitro. Comparing with traditional measurement indicator such as aggregation activity and HSR for platelet function evaluation, it take longer time and need the following in vivo experiment validation, To use CEIFI as function measurement indicator, this method still need a large number of clinical studies data support. From our experiment, the results showed some possibilities of being a potential indicator for the detection of overall platelet state and functional integrity.
